# Data Analytics, Self-Organization, and Security Provisioning for Smart Monitoring Systems

**DOI:** 10.3390/s22197201

**Published:** 2022-09-22

**Authors:** Raja Waseem Anwar, Kashif Naseer Qureshi, Wamda Nagmeldin, Abdelzahir Abdelmaboud, Kayhan Zrar Ghafoor, Ibrahim Tariq Javed, Noel Crespi

**Affiliations:** 1 Faculty of Computer Studies (FCS), Arab Open University, Muscat P.O. Box 1596, Oman or; 2 Department of Electronic & Computer Engineering, University of Limerick, V94 T9PX Limerick, Ireland or; 3 Department of Information Systems, College of Computer Engineering and Sciences, Prince Sattam bin Abdulaziz University, Al-Kharj 11942, Saudi Arabia; 4 Department of Information Systems, College of Science and Arts, King Khalid University, Muhayil Asir 61913, Saudi Arabia; 5 Department of Computer Science, Knowledge University, University Park, Kirkuk Road, Erbil 446015, Iraq; 6 Center of Excellence in Artificial Intelligence (CoE-AI), Department of Computer Science, Bahria University, Islamabad 44000, Pakistan; 7 Institut Polytechnique de Paris Telecom SudParis Evry, Courcouronnes FR, 9 Rue Charles Fourier, 91000 Evry, France

**Keywords:** IoT, security, 5G, networks, big data, analytics, communication, quality of services, SVM, decision tree, KNN, DDoS, environmental monitoring

## Abstract

Internet availability and its integration with smart technologies have favored everyday objects and things and offered new areas, such as the Internet of Things (IoT). IoT refers to a concept where smart devices or things are connected and create a network. This new area has suffered from big data handling and security issues. There is a need to design a data analytics model by using new 5G technologies, architecture, and a security model. Reliable data communication in the presence of legitimate nodes is always one of the challenges in these networks. Malicious nodes are generating inaccurate information and breach the user’s security. In this paper, a data analytics model and self-organizing architecture for IoT networks are proposed to understand the different layers of technologies and processes. The proposed model is designed for smart environmental monitoring systems. This paper also proposes a security model based on an authentication, detection, and prediction mechanism for IoT networks. The proposed model enhances security and protects the network from DoS and DDoS attacks. The proposed model evaluates in terms of accuracy, sensitivity, and specificity by using machine learning algorithms.

## 1. Introduction

The environmental section has suffered from various issues and challenges related to air quality, clear and drinking water availability, and pollution [1,2]. Two of the significant requirements of a human being are clean and fresh air and water. Environmental monitoring systems provide and control environmental factors by using smart technologies and atmosphere monitoring, controlling other factors, such as waste, pollution, and recycling processes. The environmental monitoring system also provides the environmental control mechanisms to measure the vital signs of the environment and is further used for any decision making. Still, there are many challenges in dealing with environmental monitoring data due to temporal dependencies, irregularities and sparsity, high-dimensionalities, and heterogeneities [3]. Furthermore, in generalized environmental data using ontologies, these challenges are more complicated. The new and integrated technologies have opened new gates for data communication. In these technologies, everyday physical objects can communicate by using connecting interfaces. The IoT is one of the areas where things are connected with the Internet with the pervasiveness of wireless technologies [4]. The main objective behind these smart networks and integration things is to simplify people’s lives and offer various smart services to protect the environment and take time in decision making. The smart home environment monitoring system is another example where users manage the temperature inside the home and control as it per their requirements. With time, IoT networks need improvement based on people’s mood or context to fit technological requirements. The boundaries between objects or things and the cyber or digital world are blurred due to overlapping characteristics [5]. Things in the form of smart devices and sensors have generated a massive amount of data and moved them to the cyber world for data analysis. The main aim of this process is to generate a suitable representation of the corresponding entities. The smart environmental monitoring system is another paradigm where things or devices offer services by using sensor nodes and other devices to monitor the vital signs of the environment, such as humidity, air pollution, water purity, carbon dioxide presence, and other pollutions factors. The IoT provides data communication facilities and connectivity by using open protocols, standards, and services. These networks generate a large amount of data that reside on the Internet [6]. In the IoT, smart sensor nodes and other devices move along with users and act as a physical world. 

Advancements and adoption of new technologies based wireless networks have gained tremendous attention due to fast data communications services. The fifth-generation (5G) technology is one of them, which is working as a bridge between data centers and other resources. The 5G technology has been adopted in smart cities and improves reliability, fast mobility, and efficiency. Due to a large number of connected devices in recent networks, the data are produced at a large scale. Data management and handling are some of the challenges, especially where all objects are connected and produce data, such as the Internet of Things (IoT), intelligent transportation systems (ITS) [7], wireless sensor networks (WSN), and smart environment monitoring systems [8]. The 5G and 6G technologies are advanced wireless network standards introduced after the 4G network. These standards are designed to virtually connect devices, objects, and machines. The 5G network provides heterogeneity in resources and communication infrastructure networks. The base station (BS) is deployed densely, where the huge amount of data traffic leads to a heavy burden on the backhaul networks [9]. This 5G technology aims to provide higher performances, ultralow latency, massive network capacity, high reliability, and high-speed services. 

According to IBM, 2.5 quintillion bytes of data are created every day, and the amount of data will be 40 trillion gigabytes in 2020, which is 44 times greater as compared with data in 2009 [9]. According to International Data Corporation (IDC), in 2020, the volume of data reached 44 zettabytes, and 10% of that data belonged to IoT networks. The data sources in 5G networks are drones, base stations, nodes, and devices [10]. Big data analytics is a process where a large amount of data are examined to uncover hidden patterns and correlate with other insights. Big data has various features, including value, variety, volume, and velocity. Variety indicates the data structure and data types, such as log files, audio, video, and smart metering reading. These data are used in different applications, such as smart grid, healthcare, and advertising. Velocity indicates the data processing and collection of data conducted in a timely manner. Volume indicates the scale of data, which is extremely big. Value indicates the wealth of the data. Different procedures are performed in big data, such as data acquisition, data processing, data transportation, and data analysis [10]. This process also improves the network performance and enhances the user’s experience, and efficiently manages the network resources, and maximizes the revenue. The big data analytics process has been successfully applied in different areas, such as industry, healthcare, and e-commerce. For example, big data analytics in a mobile cellular network is collecting separate data and understanding the behavior of users from multiple perspectives. Another advantage of big data analytics is real-time processing in which an operator can monitor the network infrastructure and make autonomous decisions [11]. These technologies continuously support wireless connectivity and fulfill the heterogonous network demand [12]. Deep learning methods also have been successfully applied for network application traffic analysis. Deep learning automatically learns the data features where it is difficult to demonstrate the data accurately [13]. There are serval types of deep learning strategies used for data traffic analysis, such as deep belief networks (BDNs), convolutional neural networks (CNNs), long short-term memory recurrent networks (LSTMRNs). Deep learning methods are helpful for big data analytics to examine the big data and provide better decision-making models and discover hidden knowledge. The processes for big data analytics are data mining, predictive analytics, machine, and deep learning. 

There are serval challenges in IoT- and 5G-based networks, such as maintenance, configuration, network planning, and optimization due to complicated usage patterns and the complexity of data traffic. Security is another significant challenge in these networks [14,15]. Data increase day by day, which need proper data privacy and security by using advanced intrusion detection mechanisms against malicious attacks. Large data complexity and a complicated IoT structure environment also cause a challenge for data collection, processing, and efficient use of a deep learning method. Big data and the 5G network are emerging as high priority for research. In this paper, we propose a data analytics model for 5G-based IoT networks and propose a concept of IoT networks for self-organizing networking to deal with big data management for smart environmental monitoring systems. This paper also introduces a security mechanism for IoT networks and designs an authentication model and detection and prediction models for IoT networks. The other objectives of this paper are as follows:

- Propose a data analytics model for 5G-based IoT networks for smart environmental monitoring systems;
- Propose a self-organizing architecture for IoT environmental monitoring systems;
- Propose a security model for 5G-based IoT networks;
- Propose a predictive model by using machine learning algorithms.

The rest of the paper is organized as follows: Section 2 presents the related works in the field. Section 3 presents the proposed data analytics and security model design phases. Section 4 presents the simulation results to check the performance of the proposed models. Section 5 concludes the paper with future direction.

## 2. Related Works

A deep-learning-based anomaly detection system for the 5G network is proposed in [14]. A deep learning method is used to analyze the network flow and cyber threats in 5G network architecture. The proposed architecture is based on a high-level management and orchestration plane. Virtualized components consist of two functions: network anomaly detection (NAD) and anomaly symptom detection (ASD). The process of accounting flow is performed in two steps, including flow collection and flow exporting. The ASD module is used to classify the different flow as normal or anonymous, then passes to the NAD module. Machine learning techniques are used for SAE and DBN. These techniques are implemented in the first level as supervised or semisupervised. The proposed mechanism is dedicated to virtualized resources and accuracy. Understanding and forecasting mobile traffic with the evolution of the 5G generation can enable intelligent management features. 

A multitasking learning (MTL) model using deep learning networks for mobile traffic analysis is proposed in [16]. The authors studied three deep learning neural networks, including recurrent neural network (RNN), three-dimensional convolution neural network (3D CNN), and a combination of RNN and CNN. The authors also customized the deep learning model by observing data traffic in the last hour. The RNN model is used to predict the future network data traffic. The 3D CNN model is used to capture the mobile traffic based on geographic-based features. A combination of RNN and CCC is used to extract the real-time features. These methods have temporal and geographical data extraction traffic features. The results indicated a 70% to 80% high forecasting accuracy of mobile data traffic and a better prediction method. 

The 5G networks are more dynamic and complex in the term of the ultradense network (UDN), where traffic demand is high. The 5G network becomes critical for using simple conventional F/TDD due to the fact that the conventional uplink and downlink configuration is the dynamic time division duplex (TDD) method. It is difficult to manage and control the network radio resources. Authors in [17] proposed a deep-learning-based radio resource assignment technique for 5G networks. The new policy for 5G UDN should be able to predict the possible congestion scenarios and eNodeB (eNB) query occupancy to manage the diverse and high demand of data traffic. This method addressed the shortcoming of dynamic time division duplex-based resource assignment in ultradense networks. It uses long and short-term memory (LSTM), a deep learning algorithm to intelligently control the 5G network resources. In this algorithm, past and current datasets are used to a localize prediction about future traffic. The proposed solution is divided into two phases, including the preparation and running phases. First, they initialized the LSTM structure and prepared the dataset from the different types of UL and DL ratios. Simulation results showed that the proposed solution improves the radio resource control in terms of the mean opinion resources (MOS), packet loss rate, and throughput. 

A self-adaptive deep-learning-based system for anomaly detection is proposed in [18]. The proposed method is based on two-level deep machine learning models. A deep learning mechanism is used to analyze the network traffic flow by extracting the features from network data flows during data communication. The proposed system is based on three modules, virtualize network function (VNF), NAD, and anomaly symptom detection (ASD) for anomaly detection in the network. The LSTM deep learning model is used for NAD. The deep belief network (DBN) and stacked autoencoder (SAE) deep learning models are adopted for anomaly symptom detection (ASD) and the time measurement process. This system allows for configuring the cyber defense architecture automatically, such as traffic fluctuation management, computing, and optimizing the resources needed for a particular moment and analysis process. The proposed system architecture is self-adapted for anomaly detection from real-time subscriber equipment’s optimization of the resource consumption.

A channel state information prediction for 5G wireless communications by using a deep learning method is proposed in [19]. This scheme is known as efficient online channel state information (CSI) prediction known as OCEAN. CSI uses historical data for prediction by using critical features, such as time, humidity, weather, frequency band, and temperature. This system also uses two algorithms for designing a learning framework, including LSTM and CNN. Further, the authors developed a two-step training mechanism for offline/online processes. The authors used four case studies, two for indoor and two for outdoor scenarios. The result showed that the OCEAN model performed accurate CSI prediction between 2.650% and 3.457%, which is the average difference ratio between the one measured and predicted by CSI. Additionally, it predicted the CSI value very quickly.

The technology of 5G applications, such as virtual reality video streaming and self-driving, requires high network performance in terms of bandwidth and low latency. The backbone Internet cannot keep up the bandwidth and application requirement in latency below 100 ms due to the physical barrier from the signal propagation speed for the store and forward the data. Mobile edge caching is one of the important factors for 5G networks. The BS is offering mobile edge computing (MEC) capabilities with strong computing modules. To bridge the speed gap by using the cashing technique effectively, MEC provides quality of services (QoS) in two aspects, including data traffic, which can be reduced by caching contents in the edge to save the Internet bandwidth, and the network latency can be reduced by caching content in the BS. A intelligent caching replacement algorithm is required to improve the edge computing in cache performance. Authors in [20] proposed smart and cooperative edge caching for 5G networks by using deep learning. They used deep learning and the deep cache method for understanding the request pattern for every BS to improve the cache decisions, in which the caching strategy automatically learns the request sequence in real time. The system uses an LSTM network for caching. The authors also trained the model by feeding raw request sequences in an online fashion. The experiment results were performed on a mobile video request dataset in a large-scale real world. The result showed that the proposed system reduces the transmission delay between 14% to 22%, and backhaul data traffic saves between 15% and 23%. 

Deep-learning-based resource allocation for the 5G wireless network is presented in [21]. First, they designed the model for resource allocation for device-to-device (D2D) communication. Second, the authors used the deep learning method to perform the optimization. The dynamic allocation of spectrum and antenna is used for generating the CNN. Channel information is used for generating the CNN to optimize the resource allocation. By analyzing the historical data of the channel condition, the authors implemented the deep learning methodology where the frequency of the pilot could be substantially reduced and the precision of the prediction was greatly improved. The proposed strategy is working very well, and experiment results indicated that the proposed scheme performance consumed less time as compared with the traditional method. 

A dynamic transmission power control to improve the efficiency of NLOS transmission is presented in [22]. In the 5G wireless communication system, this method maximized the total data rate, which was reached by all UEs. The authors explored the control of user equipment (UE), which is associated with microcell BS or small-cell BS (MBS/SBSs). The power allocation is used to optimize the sum rate of UE transmission power constraints and the QoS of UEs. The authors proposed a deep Q-network (DQN), which is based on reinforcement learning but uses a deep neural network to predict the Q-function. Q-function is estimated offline and carried out online to derive the control strategy. Therefore, the UE associations with power allocation and MBS/SBSs significantly reduce the computation time. The results indicated that the proposed scheme has improved the sum rate. The sum rate decreases with the increase in the minimum signal-to-interference-plus-noise-ratio (SINR) requirement. A higher transmission power needs to be allocated to some links where other links are allocated to a lower transmission power, and the sum rate is decreasing as a result. 

Optimized BS allocation for platooning vehicles by using a deep learning algorithm based on 5G-V2X is presented in [23]. Allocating a 5G BS is used for platooning vehicles to create an artificial intelligence model. The 5G network technology can update vehicular communication from C-V2X to 5G-V2X. A large BS uses low frequency, and a small BS uses high frequency to eliminate dead angle and expanded bandwidth. It is challenging to choose the most suitable BS. The proposed method is divided into three parts: highway and platooning vehicles, designing a network simulation environment, and reinforcement learning for BS allocation. The authors used a deep Q-network reinforcement learning method to train the model for optimizing a suitable BS allocation. Whenever a platoon vehicle is selected, the BS is used as reward value for the DQN for a continuous artificial intelligence model. From these techniques, the authors achieved an optimized BS allocation.

An identity management and authentication scheme for IoT networks called YubiAuthIoT proposed in [24]. The proposed scheme generates a token for device identity, where devices are connected to a centralized system for trust evaluation. The trusted pools are created based on trust values to authenticate devices. Ephemeral keys are used for clients, where managers use a secure token by using key storage. The stolen and broken issues are also resolved by using a personal identification number for key access. This solution provides a decentralized solution as well for device management. The proposed solution is tested with a firmware platform where different IoT devices are used with security functionality. After completion of these processes, the IoT devices communicate with each other and encode the http requests. However, due to IoT devices’ limited features, the complex process degrades the network performance and causes delay and overhead in the network. 

A quality of services is aware of a scheme for an IoT network by taking the Sybil detection mechanism proposed in [25]. The proposed scheme identified the Sybil nodes and their forged identities for multihop data communication. When the proposed system detected the Sybil nodes in the network, then an optimal contention window was selected for quality provisioning. Basically, in traditional networks, the medium access control layer performs data throughput and fairness, but when the nodes generate large data, this strategy fails. The proposed scheme is a lightweight solution, which has two phases, including the Sybil node detection and then the determination of the optimal contention window size. The simulation results indicated the better performance of the proposed scheme in terms of quality of service parameters. However, there are various attacks existing in IoT networks, and only Sybil attack detection is not enough to evaluate the overall network performance. 

## 3. Proposed Data Analytics Model for 5G-Based IoT Networks

**Figure 1:**
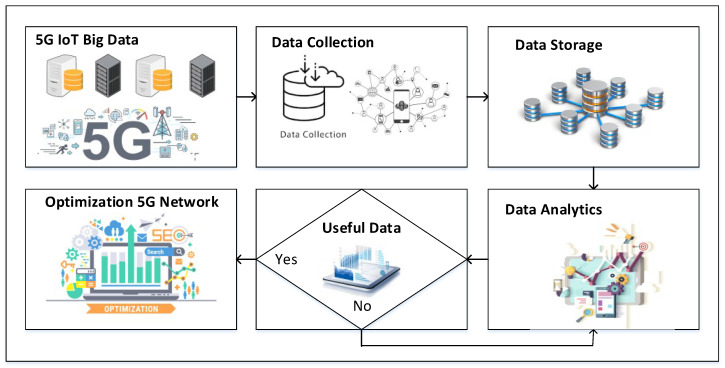
Proposed data analytics model.

The growth of smart devices causes the current cellular communication system overload due to the increase in mobile data traffic. The current wireless communication infrastructure is shifting to next-generation 5G technologies to handle the large big data and utilize serval technologies to improve its system capabilities. Non-line-of-sight (NLOS) transmission is used everywhere, and it is also more common in the 5G wireless communication system due to the use of millimeter-wave (mmWave) communications. The proposed big data analytics model is based on data collection, data preprocessing, and data analytics. Data collection is one of the significant components and processes. Data collected from 5G-based IoT networks is from different external and internal sources. The external data are gathered from service areas, organizations, market, and complaint departments. The internal sources are operating systems, supporting systems, and business systems. In the proposed model, collected data are divided into two categories, including data sources and auxiliary tools. The IoT devices are data collection tools and are able to collect multimedia data by using sensor nodes, cameras, and GPS systems. These devices are equipped with Wi-Fi and Bluetooth technologies. The network data are collected by using some packet capturing applications, such as SmartSniff and ComView. After data collection, the next step is data preprocessing, including data storage. Data storage is one of the complex tasks due to its massive and diverse properties. After data storage, the next step is data analytics by using different machine learning methods. Data analytics optimizes the data and generates useful information. This process provides data efficiency and meets the requirements. The flow diagram of the proposed data analytics model is shown in Figure 1. 

### 3.1. Data Analytics

In data analytics, the collected data from customers and networks are analyzed to uncover the data patterns and determine the customer’s and subscriber’s data who left the services due to service issues in the 5G-based IoT network. This model also deals with the user’s data in terms of device behavior and services. Machine learning methods are adopted to analyze the data. The data analytics model has three main modules, including data analysis, data training, and evaluation and prediction. In the data analysis module, we check the relationship between predictors. This step is more helpful to establish the interrelationship between variables and is also used to observe the behavior of the variables. In the data training phase, the collected dataset is divided into partitions and an applied machine learning algorithm on the training set. Various types of algorithms have been adopted to train and predict the features. The selection of the machine learning method is another important factor to target the predictive variables. We adopted tree-based methods, including classification tree, random forest, and boosting models. The last phase is model evaluation and prediction, where we will evaluate the proposed model performance and predict the testing data.

### 3.2. Proposed Self-Organizing Architecture for Smart Environmental Monitoring Systems

The concept of self-organizing architecture is introduced because the existing IoT networks are not able to handle the device-centric and self-organizing networks. In these networks, the users are an active element of services and applications. Personal devices are key nodes for decision-making and data dissemination functions. These networks use the resources and optimize the system operations. The main concept of these networks is to design a platform where users solve their complex problems in a synergic way through computers [26]. We proposed a self-organizing architecture by using smart devices and sensor nodes where the devices learn the environmental changes and user behavior. The sensor nodes learn the user’s daily activities related to home atmosphere, car atmosphere, air purity, and pollution in the surroundings. The smart devices automatically monitor all the environmental factors without device interaction. The smart devices behave in a self-organizing manner and generate alerts for any possible environmental threat. The proposed architecture is based on three layers, including the environmental monitoring systems layer, communication layer, and IoT layer. In the first layer, the different service-oriented components are used for device registry, application management, and repository and action management, especially for environmental monitoring systems. 

**Figure 2:**
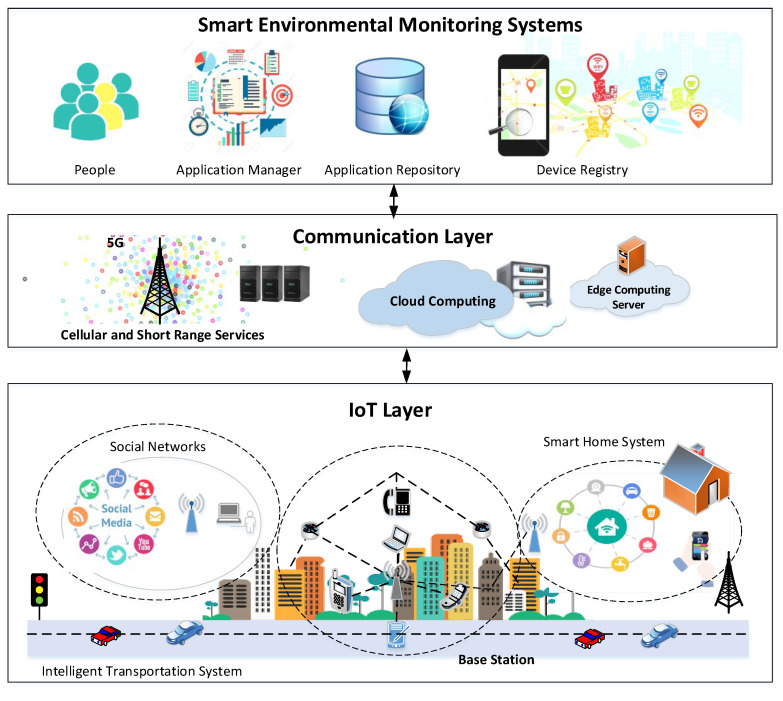
Proposed self-organizing IoT architecture.

In the first layer, the smart devices work as central components to maintain information related to the system manager. These devices work to collect environment details, such as air quality, pollution, atmosphere, temperature, and other vital signs for gases or emission systems. In this layer, the smart devices work as a companion device and are able to interact with surrounding devices, such as car navigators and smart homes. The registry services provide the processes related to property change. This layer handles all types of environmental data, where the smart devices maintain information related to the surrounding monitoring or device information. Smart devices provide an interface to people, where they check the pollution level of routes and traffic situation in the user routing route. The devices also observe the user’s daily routines and medical conditions, which are compared with sensed environmental signs. Table 1 shows the self-organizing activities and their integration with IoT networks, whereas Figure 2 shows the proposed self-organizing IoT architecture.

### 3.3. Proposed Security Model for IoT Networks

The proposed security model is based on three modules: authentication, malicious node detection, and prevention. The proposed model is deployed on edge devices to monitor the 5G-based IoT networks. This model is designed for unauthorized nodes in IoT networks and copes with the inaccurate information generated from the malicious nodes. In the first module, the proposed model deals with illegitimate devices by using an authentication mechanism. The second module is designed for detection and prevention methods to deal with malicious node detection and prevention mechanism. The complete design of the proposed model is explained in the next subsections.

#### 3.3.1. Authentication Module

**Figure 3:**
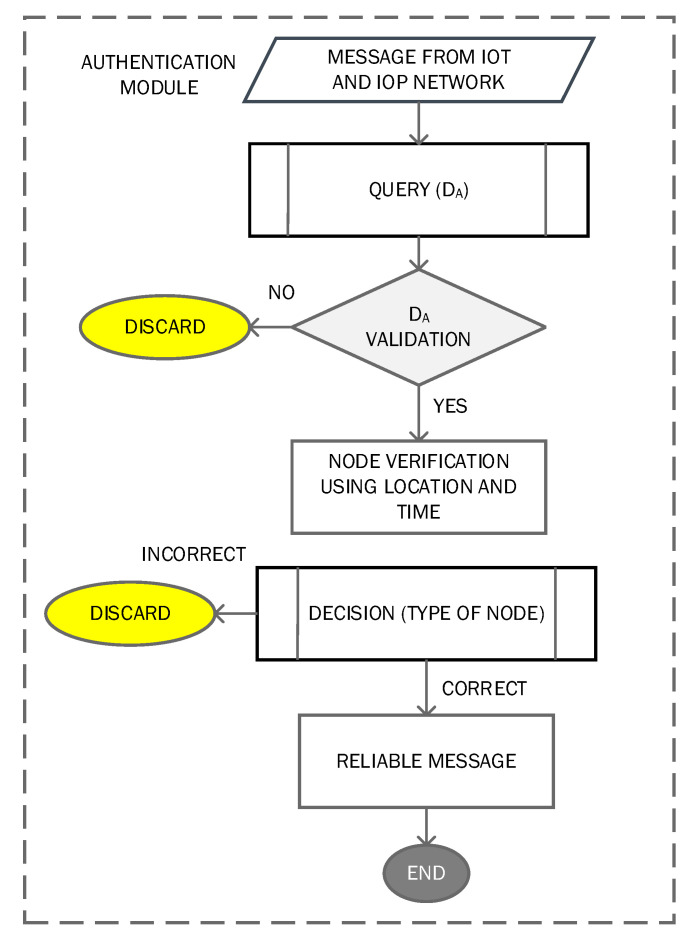
Authentication module process.

This module provides authentication services for reliable and secure data communication in IoT networks. The authentication method is one of the significant methods for security, and it also ensures the integrity of nodes in terms of accuracy of any event message. The edge network devices are used to authenticate the network nodes because these devices have more capabilities in terms of processing power, memory, high throughput, and less latency. Every device in the IoT network is registered with an edge device that has a complete list of nodes that are within its defined range. The unauthorized nodes produce inaccurate data threats for the network. We proposed an authentication method for edge devices. We assumed that the edge devices have all the information related to registered devices in IoT networks. For the authentication mechanism, the proposed module accesses the authentication of IoT devices denoted with di by dj and a query from the relevant edge node by dj. At the edge devices’ side, a large number of data are collected from IoT devices. The traditional data structures are not suitable to handle big data. To address this, the proposed authentication module uses a probabilistic data structure to reduce the latency and improve the analytical process. For data analytics, the common space-efficient probabilistic methods are cuckoo filter (CF), bloom filter (BF), and quotient filter (QF). These methods have been adopted for massive datasets and are better for checking whether an element is a member of the dataset or not. The QF provides a better querying structure and is better in terms of secondary memory. On the other hand, the CF is easier for implementation than others and uses less space as well. However, the cuckoo method filter is better than others due to its higher throughput, which is one of the requirements of 5G-based IoT networks. CF is one of the compact variants that store only fingerprints rather than key values. The fingerprints refer to a method that uses only a hash function for every inserted item. The hash function provides a method for data storing in an array format and has individual index value. This method is also one of the open-addressing methods for constant lookup in the worst case. By using the partial key in the cuckoo, the hash table achieves compatibility and high utilization when the fingerprints are stored. The hash table contains the data related to the mode in an array format and has an individual index value for the data value. Cuckoo hashing is one of the alternate methods for constant lookup in the worst case. Figure 3 shows the authentication module process. 

#### 3.3.2. Detection and Prediction Model

Due to the number of connected devices, malicious nodes are always threats to users. The proposed module uses a detection mechanism for node detection. This module checks the malicious nodes and works as an intrusion detection system (IDS). The proposed detection and prevention module monitors the network and detects the threats coming from the network. The main components of the proposed IDS system are packet capturing, feature extraction, packet preprocessing, classification, and event detection. In packet capturing, the system is working on the live mode by using an open-source library. JnetPcap is used for packet capturing coming from the IoT network and decoding the packets. After this phase, the next step is feature extraction by using the PcapWT as proposed in [27]. This tool provides fast packet lookup by indexing original traces using a wavelet tree structure. The extracted feature from packets is around 50. We used a dataset based on DDoS attacks called CICDDoS2019 [26]. This dataset is based on real-time DDoS attacks, including DNS, MSSQL, NetBIOS, UDP, and TFTP. This dataset has around 90 features to train the machine. For new features, we adopted the Pcap analysis method. After feature selection, the next step is data preprocessing for data structure, cleaning, and organizing the data for machine learning models. The data are divided for testing and validation purposes, such as 70% for training, 20% for validation, and 10% for testing. Data normalization is performed to avoid the influence of features with high values. Equation (1) shows the normalization calculation.

(1) $$nv=\frac{ov-\min\left(o\right)}{\max\left(o\right)-\min\left(o\right)\;}$$

The ov denotes the old value of the extracted feature, where $\min\left(o\right)$ shows the minimum value of packets. The $\max\left(o\right)$ denotes the maximum value of the feature in packets, and *nv* shows the new normalization value. After the normalization process, the values of the dataset fall between 0 and 1. 

**Figure 4:**
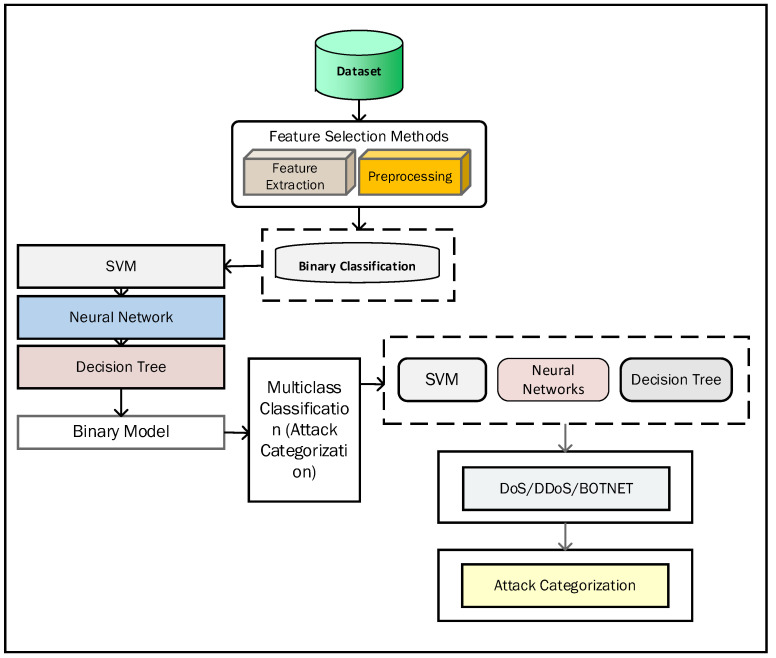
Training model architecture.

For classification, we adopted machine learning algorithms called SVM, decision tree, and artificial neural network (ANN) to create the model. The ANN is one of the well-known methods of deep learning inspired by brains and their behavior. In the ANN, the multiple nodes are connected with each other for data classification. The ANN is widely used for anomaly-based systems to design the model and find the unknown relationship between various parameters in the dataset. The SVM is one of the machine learning methods used for classification and is adopted in the proposed model. The SVM is a supervised machine learning method used for the classification of two class problems. This method is adopted for DDoS attack detection based on classification. We also adopted a decision tree for classification and regression problems. In this method, the rules are displayed as rows and conditions as columns and create a treelike structure, where each feature of the dataset is represented by each node of the tree. For two-class classification, a model is trained and used as a prediction model. First, we used the dataset and applied preprocessing on it and then achieved a binary model and obtained binary classification and trained the model. We achieved the attack categorization model, which is used for prediction. Figure 4 shows the architecture diagram of the proposed model.

**Figure 5:**
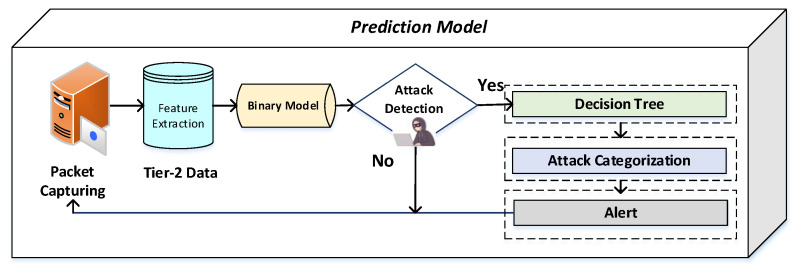
Proposed predictive model.

After training a model design, the DDoS detection model designs for real-time prediction. First, the packets are captured from network flows from edge devices by using device network interface cards. After packet capturing, the next step is feature extraction from captured packets, which are normalized and sent to a binary model for classification. In the classification, the malignant traffic and benign traffic are evaluated, and attacks are sent for attack classification, and an alert will be generated. Figure 5 shows the predictive model process. 

#### 3.3.3. Adopted Algorithms

For the classification, we used three machine learning algorithms, including SVM, decision tree, and ANN. These algorithms are used to examine the proposed model and classification of DDoS attacks. Various other methods have been adopted for classification, but the SVM is one of the well-known approaches especially when the data are in unstructured and semistructured form. This method has a high scale relatively for high dimensional data. The second adopted method is the decision tree, which is another good option for a possible decision outcome. This method is used for comprehensive analysis and is able to identify the decision nodes. 

The SVM is one of the supervised learning models to label one or two classes or strata. The SVM trained and classified with new samples points in space and arranges the samples into separate classes by using a hyperplan, where the new samples are mapped into one of the classes [28]. The SVM is also useful for classifying the boundary to separate the input vector into one or two classes. The second algorithm is the decision tree, which is used as an analytical and pictorial tool. A decision tree uses two shapes, oval and rectangle, to represent the decision nodes and the class label node. The decision tree also has decision rules, where the class label is linked to a decision node [29,30]. The ANN is one of the simple and effective methods for classification. This is a nonparametric classification approach where the data record is classified with the nearest neighbors. This classification is used without consideration of distance-based weighting. To apply the ANN, we need to select an appropriate value for k, and the success is based on this value. This method is case based, where all the training data are kept for classification. With some benefits, this method is lazy and not useful for web mining for large repositories. This method is more useful for text categorization to improve efficiency and classification accuracy. 

## 4. Experimental Results

In this section, the complete results of the SVM, decision tree, and KNN models are discussed with comparative analysis based on the classification of the DDoS dataset. The results are evaluated based on accuracy, sensitivity, and specificity. 

Feature selection results are performed by using various classifiers. As discussed in the above section, a dataset is divided into two main parts, training and testing the dataset, and examines its performance variation. Different experiments are performed to evaluate the proposed model performance in terms of model accuracy. The first experiment is conducted for registry feature extraction from memory analysis. In the first experiment, we check the feature selection to select the best features from the dataset. Feature selection is used to enhance the accuracy. We use a univariate approach to identify the significant features of the dataset. The feature selection is based on specific criteria and based on high scores and rank. We select the best k features, where we have the better results; for example, for 50 features, the result is 0.95, and for 100, the result is 0.98. The features’ importance result shows that the plot_n is the most important feature on a normalized scale where the sum of the features to 1. It also indicates the cumulative feature importance versus the number of features. 

### 4.1. Accuracy Analysis

For this analysis, we used three classification models, including SVM, decision tree, and ANN. Accuracy provides the ratio between total instances examined and total correctly identified instances in a dataset. Accuracy is compared against every algorithm in each data set. The proposed predictive model denotes P4 given a problem instance to be classified using a combination of multiples from the three most famous predictive models. Mathematically, a proposed predictive model can be formulated as follows:

$P_{j}$= is the predictive models (criteria), where $j=\left\{1,\ldots ,p\right\}$ and p is several predictive models.

P1: SVM
P2: decision tree
P3: KNN model

The accuracy performance is calculated by using $Acc_{j}$ which denotes the accuracy of predictive models and $P_{j}$ is based on Equation (2):

(2) $$Acc_{j}=\left(\;\sum_{i=1}^{n}\mathrm{TP}\;\left(i\right)\right)+(\sum_{i=1}^{n}\mathrm{TN}\;\left(i\right))\;/(\sum_{i=1}^{n}\left(\mathrm{TP}\;\left(i\right)+\;\mathrm{FN}\;\left(i\right)+\;\mathrm{FP}\left(i\right)+\mathrm{TN}\;\left(i\right)\right)$$

where *n* is the number of the outcomes and 1 ≤i≤n .

The accuracy results are tabulated in Table 2. 

**Figure 6:**
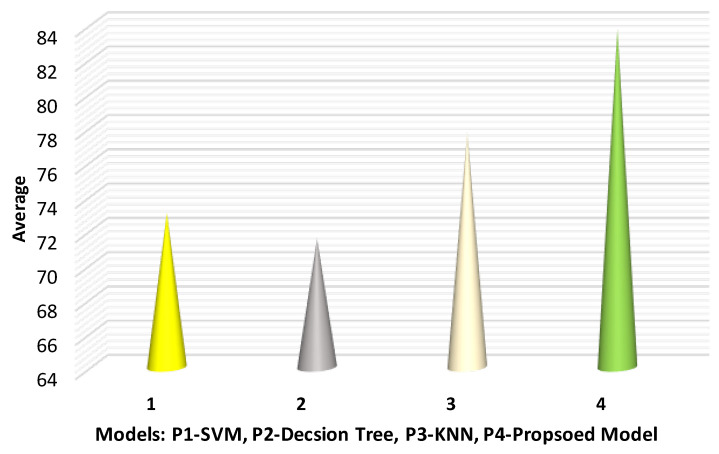
Average accuracy analysis with different models.

The result shows the average ranking of the obtained accuracy results of the proposed model with the other three models by using the Friedman test. The result indicates that the proposed model has improved results as compared with the others in terms of accuracy. Additionally, this experiment rejected the null hypothesis where all the accuracy results are equal. On the other hand, the 2-decision tree predictive model has the worst average rank among other predictive models. P1-SVM and P3-KNN have better results as compared with P2-decision tree. Figure 6 shows the accuracy results. 

### 4.2. Sensitivity Analysis

Sensitivity analysis is presented here to check the proposed model sensitivity average as compared with the other three models, including SVM, decision tree, and KNN. The dataset is evaluated to predict the outcome and then validated in terms of sensitivity by using Equation (3).

(3) Sensj=(∑i=1nTP i)(∑i=1n(TP i+FNi))

The comparison is shown in Table 3 of the proposed prediction model and the other three models.

**Figure 7:**
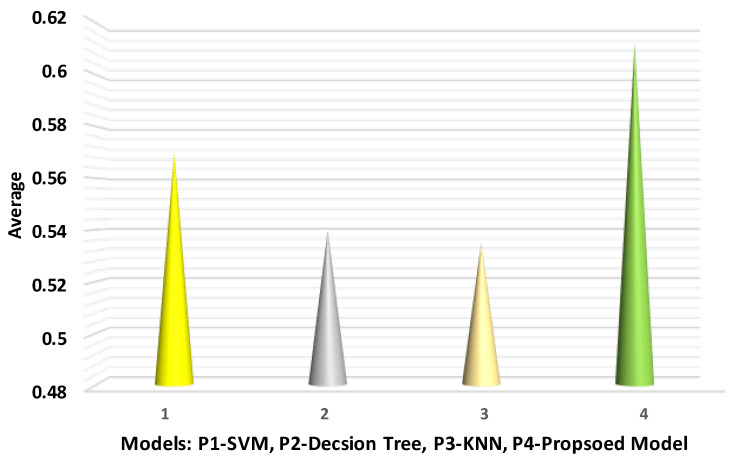
Average sensitivity analysis with different models.

The results indicate that the proposed model has better results as compared with the existing models in terms of sensitivity. P4 achieved the best average by using Friedman statistics compared with P1-SVM, P2-decision tree, and P3-KNN models. In this test, P3 obtained the worst average because KNN is a lazy method and has low efficiency. Figure 7 shows the sensitivity results. 

### 4.3. Specificity Analysis 

The specific results show the proposed and the other three models, SVM, decision tree, and KNN predictive models. The dataset is used to predict the outcomes for validation, and the 4-fold is used for specificity calculation. Equation (4) presents the specificity calculation, and Table 4 shows the results.

(4) $$Spec_{j}\;=\left(\sum_{i=1}^{n}\mathrm{TN}\;\left(i\right)\right)/\left(\sum_{i=1}^{n}(\mathrm{FP}\;\left(i\right)+\mathrm{TN}\left(i\right)\right))$$

**Figure 8:**
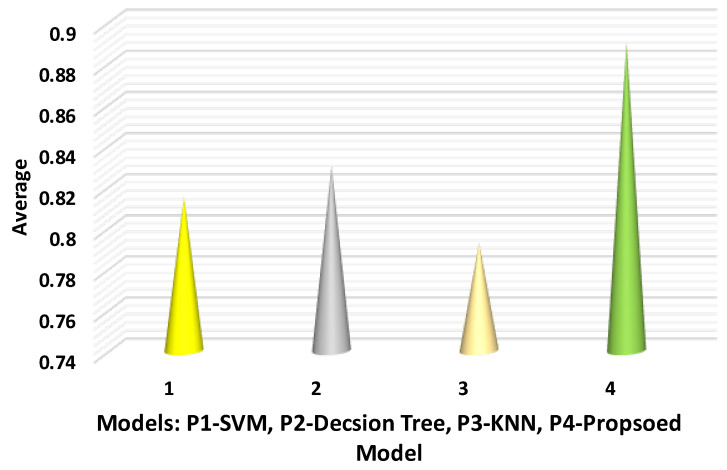
Specificity analysis.

The results indicate that the proposed model has better results as compared with the other three models in terms of specificity. P5 achieved the best average by using Friedman’s statistics compared with the P1, P2, P3, and P4 models. In this test, P3 obtained the worst average because KNN is a lazy method and has low efficiency. Figure 8 shows the sensitivity results.

First of all, the results are evaluations of the proposed dynamic and predictive model through SVM, decision tree, and KNN. We used a dataset based on DDoS attacks called CICDDoS2019 [26]. This dataset is based on real-time DDoS attacks, including DNS, MSSQL, NetBIOS, UDP, and TFTP. The noise reduction and data normalization methods are already applied to the database to avoid noise and missing values. The SVM, decision tree, and KNN are trained and configured based on selected features. The results indicated that the proposed predictive model achieves significant results and the best average ranking in terms of specificity, accuracy, and sensitivity. The proposed predictive model is helpful in determining the malware detection in IoT networks and is able to access the damage caused by intrusive actions and malware. The proposed model is also helpful in identifying the level of difficulty and complexity in malware.

## 5. Conclusions

Internet evaluation and connected devices have created new concepts, such as the IoT network, based smart environmental monitoring systems, where people and sensor devices are the key elements of the network to sense the environment data, such as pollution, air quality, and atmosphere. Due to the big data generation and open nature of these networks, data handling, self-organization, and security are always a challenge. The IoT network concepts and architecture are still in a blur situation, where the standards and technologies need to be revised and developed. In this context, this paper proposed three models, including the data analytics model, self-organizing model, and security model. In data analytics, big data analytics is performed on data collection, by using data preprocessing and data analytics. A self-organizing model is also proposed by using smart devices, where the devices learn the user behavior. The smart devices learn the user’s daily activity and usually use route and time. The smart devices automatically monitor all the user activity without device interaction. The security model is also presented in this paper based on authentication, detection, and prediction mechanism. The DDoS attacks dataset is adopted to test the proposed predictive model in the presence of other machine learning algorithms. The proposed model is evaluate in terms of accuracy, sensitivity, and specificity by using machine learning algorithms and showed better performance. In the future, we will consider more attack datasets and try to evaluate the model with more machine learning models. This work will be implemented in other areas and networks.

## Figures and Tables

**Table 1:** Self-organizing activities and IoT.

S#	Activity	IoT Network Integration
1	Air quality	Collect the daily air quality values and analyze the atmosphere
2	Car navigation	Collect the daily route and car atmosphere information and suggest better precautions to avoid a more polluted route by using real-time traffic systems
3	Weather alerts	Collect the weather conditions and atmosphere condition and alert people about the latest information
4	Smoke area indication	Alert the user about surrounding smoking zones based on sensed data and historical analysis about the place

**Table 2:** Accuracy of the proposed predictive model based on the DDoS dataset.

Method	Fold-1	Fold-2	Fold-3	Fold-4	Average
P1 (SVM)	77	71	74	70	73
P2 (decision tree)	69	71	72	74	71.5
P3 (KNN model)	75	76	79	81	77.75
P4 (Proposed model)	80	83	85	87	83.75

**Table 3:** Sensitivity of the proposed predictive model.

Method	Fold-1	Fold-2	Fold-3	Fold-4	Average
P1 (SVM)	0.62	0.63	0.63	0.4	0.57
P2 (decision tree)	0.55	0.51	0.6	0.5	0.54
P3 (KNN model)	0.53	0.5	0.53	0.58	0.535
P3 (proposed model)	0.61	0.62	0.62	0.6	0.6125

**Table 4:** Specificity of the proposed predictive model with other models.

Method	Fold-1	Fold-2	Fold-3	Fold-4	Average
P1 (SVM)	0.83	0.82	0.81	0.8	0.815
P2 (decision tree)	0.84	0.81	0.86	0.81	0.83
P3 (KNN model)	0.82	0.78	0.81	0.76	0.7925
P4 (Proposed model)	0.88	0.89	0.91	0.88	0.89
